# Optimal Channel Selection of Multiclass Motor Imagery Classification Based on Fusion Convolutional Neural Network with Attention Blocks

**DOI:** 10.3390/s24103168

**Published:** 2024-05-16

**Authors:** Joharah Khabti, Saad AlAhmadi, Adel Soudani

**Affiliations:** Department of Computer Science, College of Computer and Information Sciences (CCIS), King Saud University, Riyadh 11543, Saudi Arabia; salahmadi@ksu.edu.sa (S.A.); asoudani@ksu.edu.sa (A.S.)

**Keywords:** brain–computer interface (BCI), motor imagery (MI), electroencephalogram (EEG), deep learning (DL), convolutional neural network (CNN), attention module, channel selection, genetic algorithm (GA)

## Abstract

The widely adopted paradigm in brain–computer interfaces (BCIs) involves motor imagery (MI), enabling improved communication between humans and machines. EEG signals derived from MI present several challenges due to their inherent characteristics, which lead to a complex process of classifying and finding the potential tasks of a specific participant. Another issue is that BCI systems can result in noisy data and redundant channels, which in turn can lead to increased equipment and computational costs. To address these problems, the optimal channel selection of a multiclass MI classification based on a Fusion convolutional neural network with Attention blocks (FCNNA) is proposed. In this study, we developed a CNN model consisting of layers of convolutional blocks with multiple spatial and temporal filters. These filters are designed specifically to capture the distribution and relationships of signal features across different electrode locations, as well as to analyze the evolution of these features over time. Following these layers, a Convolutional Block Attention Module (CBAM) is used to, further, enhance EEG signal feature extraction. In the process of channel selection, the genetic algorithm is used to select the optimal set of channels using a new technique to deliver fixed as well as variable channels for all participants. The proposed methodology is validated showing 6.41% improvement in multiclass classification compared to most baseline models. Notably, we achieved the highest results of 93.09% for binary classes involving left-hand and right-hand movements. In addition, the cross-subject strategy for multiclass classification yielded an impressive accuracy of 68.87%. Following channel selection, multiclass classification accuracy was enhanced, reaching 84.53%. Overall, our experiments illustrated the efficiency of the proposed EEG MI model in both channel selection and classification, showing superior results with either a full channel set or a reduced number of channels.

## 1. Introduction

A motor imagery-based brain–computer interface (BCI) is the most commonly used paradigm. The use of this system facilitates the communication between humans and machines [1,2]. In most cases, research involves recording neural activity on the scalp using non-invasive electroencephalography (EEG), as it is a practical and inexpensive method [3]. An EEG signal derived from motor imagery (MI) is generated when a subject visualizes a movement without actually performing it. During motor imagery, specific brain regions are activated similar to those engaged during physical movement, primarily within the sensorimotor cortex. EEG records this neural activity by detecting fluctuations in electrical patterns across the scalp. These fluctuations manifest as distinct patterns in the EEG signals, particularly in the alpha (8–13 Hz) and beta (13–30 Hz) sub-bands. Specifically, decreases in signal amplitude, known as Event-Related Desynchronization (ERD), occur in regions opposite the imagined movement, while increases, termed Event-Related Synchronization (ERS), appear in areas adjacent to the movement side. Such capabilities allow EEG to effectively monitor brain activity in real-time, which is vital for applications like brain–computer interfaces (BCIs) [4]. MI-EEG-based BCIs aid in rehabilitation activities for individuals with disabilities and enable them to perform everyday tasks more autonomously by controlling external devices, such as robotic prosthetics or computer interfaces.

Even with extensive research focusing on MI-EEG-based BCIs, there are still many challenges to overcome. EEG signals have a low signal-to-noise ratio (SNR) where the data could be corrupted by any artifact such as eye movements. Moreover, EEG signals are subject to non-stationarity issues, which means that they may vary considerably between trials or even within the same trial for the same subject [4]. Consequently, the variability and complexity of individual brain signals during motor imagery tasks make it difficult to develop a model that can be applied universally. Moreover, EEG signals contain redundant channels that may impact accuracy and efficiency in MI task classification. These channels carry information about background neural activity. However, some channels contain redundant information and require more calculation to be, efficiently, detected and sorted out [5]. Thus, we believe that an appropriate channel selection would contribute to improving the accuracy and reducing the computational time and complexity. However, achieving such an objective of channel selection has to be carefully performed due to the drastic impact that this action has on the accuracy of MI task detection [3]. This paper addresses this issue. It proposes a new method based on a combination of channel selection and classification techniques for efficient MI task detection.

In terms of classification methods, traditional machine learning (ML) and deep learning (DL) are the two main approaches used. The majority of ML approaches have been based upon common spatial patterns (CSPs) including the filter bank CSP (FBCSP) or regularized CSP (RCSP) to extract signal features. These achieve a good performance after being followed by a Support Vector Machine (SVM) and Linear Discriminant Analysis (LDA) classifiers [5,6,7,8,9,10]. However, the ability of DL methods to extract features from raw data has made DL methods increasingly popular in recent years in BCI studies. There has been considerable attention paid to DL for its excellent performance in a variety of fields, such as image classification, speech recognition, and text analysis [11]. As DL techniques have been successful in other fields and can be utilized for automatic feature extraction, there is a strong motivation to apply them to EEG data analysis and classification. Convolutional neural networks (CNNs) are the most common model used for extracting temporal and spatial features from MI EEG data [12,13]. Several CNN architectures have been proposed as a baseline of EEG signal classification with the ability of channel and spatial classification relying on the nature of fast training and few parameters used; such models are ShallowNet [14], DeepConvNet [14], and EEGNet [15]. Many papers provided a lot of attention in order to improve the performance of these lightweight CNN models by applying them to diverse frameworks with many types of extra blocks. The structure still needs more enhancement to have the ability to classify any type of EEG data. Despite the advancements brought about MI-EEG data by DL techniques, it should be noted that these methods often struggle with high variability in signal quality across different subjects, which can severely impact classification accuracy. This highlights that current techniques remain insufficient and require further development. The attention mechanism is another aspect of architectural design used to draw attention to the most important features gained from the neural network model. Utilizing attention-based feature optimization is useful to enhance the representation power of the CNN model with minimal overhead [16,17].

Based on the channel selection process, when selecting the optimal channels, it is possible to improve the classification results while ensuring that no significant channels are removed [3]. Due to this, the majority of researchers [18,19,20,21,22] selected different channels for each subject individually. Selecting a fixed channel for all subjects is a problem that has not previously been addressed. Therefore, in order to select a fixed optimal channel for all subjects while maintaining all significant channels, a novel technique must be developed.

For the purpose of responding to these concerns mentioned above and to increase the performance of lightweight neural networks, this paper proposes a multiclass MI classification based on a Fusion convolutional neural network with Attention blocks (FCNNA) and channel selection. The main contributions of this work are summarized as follows:

- Propose a CNN structure that contains two layers of convolutional blocks followed by CBAM attention methods concatenated to better classify two classes and four classes of preprocessed EEG raw data.
- Evaluate the classification performance on a publicly available dataset utilizing two strategies: within-subject strategy and cross-subject strategy. According to our experiments in multiclass and two-class classification, our model exhibits significant improvements over existing state-of-the-art approaches.
- Propose a channel selection mechanism that maintains the performance of the proposed model with less computation cost. In this study, a novel technique is employed to introduce a fixed set of channels for all subjects alongside a variable set of channels.
- Illustrate the enhancement in performance that results from adding channel selection to our model. Moreover, a comparative analysis with state-of-the-art methods is applied which demonstrates an improvement.

The rest of the paper is organized as follows: Section 2 reviews the current DL classification techniques employed in EEG signals and briefly describes various channel selection algorithms. A detailed explanation of the methods and architectures proposed in this study is provided in Section 3. In Section 4, the experimental results are presented and discussed. The paper is concluded in Section 5.

## 2. Related Works

### 2.1. EEG Signal Deep Learning Classification

**Figure 1:**
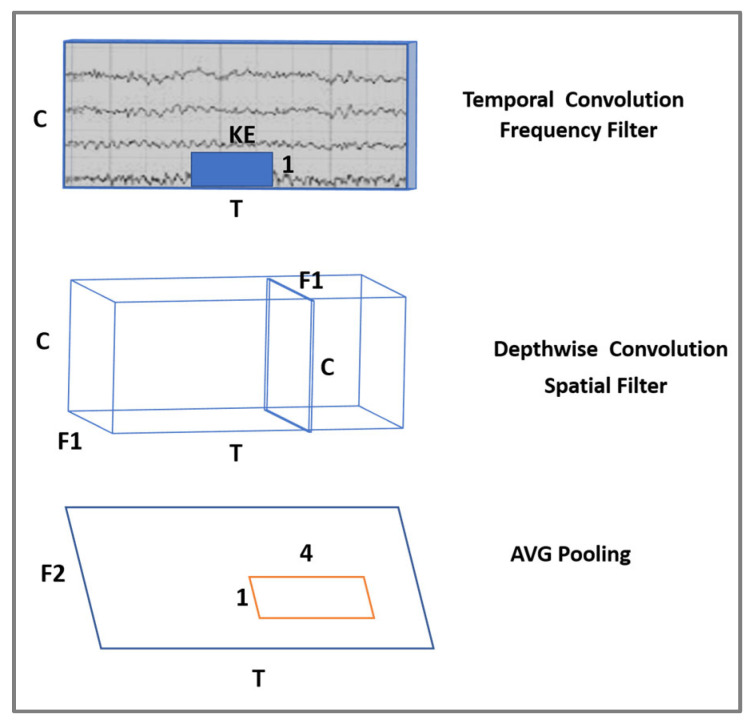
ConvNet structure. C = number of channels; T = number of time points; KE = kernel width; F1, and F2 = number of filters.

The authors in manuscript [14] proposed a ConvNet structure to design two CNN models: ShallowNet and DeepConvNet models. Figure 1 demonstrates the structure of ConvNet which combines two main layers in one block which is the first CNN block. These two layers intend to handle the channels of EEG data in two ways, first by gathering the data of one channel in a specific range of time (temporal convolutional layer), and the second layer is merging all the data of all channels in this specific time (spatial convolutional layer). In light of this description, the purpose of developing ConvNet is to become a general tool for decoding brain signals. Later, Lawhern et al. [15] introduced EEGNet, a more compact and efficient CNN architecture with few parameters and fast training nature. EEGNet enhances the idea of the ConvNet structure provided by [14] to improve accuracy and efficiency. EEGNet showed encouraging results on several types of EEG datasets with significantly fewer parameters than ShallowNet and DeepConvNet. In addition, the simple architecture of EEGNet has made it a notable candidate for EEG analysis in different scenarios.

ShallowNet, DeepConvNet, and EEGNet have yielded many other techniques that may be used as benchmarks for comparison and improvement [4] such as EEG-TCNet [23], MSFBCNN [24], and TCNet Fusion [25]. In their publication [23], the authors introduced the EEG-TCNet model, which integrates the feature extraction layers of EEGNet with the temporal convolutional network (TCN). The TCN effectively utilizes temporal information through the implementation of two layers of residual blocks. Consequently, the authors presented two models; the first with a consistent set of hyperparameters for all subjects achieved an accuracy of 77.35%, while the second, incorporating subject-specific hyperparameters, attained a higher accuracy of 83.84%. The authors in [24] introduced a parallel multiscale filter bank convolutional neural network, employing four temporal convolutions with distinct kernel sizes. These convolutions are concatenated and applied to spatial convolution, resulting in an accuracy of 75.12%. Ultimately, the researchers in [25] executed a CNN model incorporating a TCN block with two stacked residual blocks. This configuration extracted additional temporal features following EEGNet, resulting in an accuracy of 83.73%.

In addition, models of multiclass MI classification published between 2022 and 2023 will be examined to compare our results with the most recent advances in the field. In [4], a MTFB-CNN model is proposed to extract diverse information from EEG data through the use of three parallel time–frequency blocks, each containing multiple one-dimensional convolutions with different kernels and scales. Subsequently, a residual network is applied, followed by the integration of a channel attention module which yields an accuracy of 84.48%. The authors in [26] developed the CMO-CNN model, which incorporates a multi-branch one-dimensional convolutional design with a Squeeze-and-Excitation network featuring two shortcut connections to create the residual block. The model was validated using two strategies, within-subject and cross-subject, achieving accuracy rates of 83.92% and 63.34%, respectively. Following the same validation strategies, the authors in [27] introduced the EEG-ITNet model, which consists of four blocks: three layers of EEGNet, temporal convolution, dimension reduction, and classification. As a result of the validation process, the model achieved an accuracy of 76.74% with a within-subject strategy and 69.44% with a cross-subject strategy. Both models exhibit high accuracy in one strategy but not in the other, indicating that neither model is universally effective across different validation scenarios. In a similar manner to [27], the authors in [28,29] modified EEGNet to improve its performance and adapted it for general use. In [28], the MBSTCNN-ECA-LightGBM model combines EEGNet layers with a channel attention module and a LightGBM classifier to achieve up to 74% accuracy for four MI tasks of different classes. By combining EEGNet and ConvNet with transfer learning, the Siamese Deep Domain Adaptation (SDDA) framework in [29] achieved 82.01% accuracy over ConvNet. According to [30], researchers developed a Subject-to-Subject Semantic Style Transfer Network (SSSTN) that utilizes Continuous Wavelet Transform (CWT) to convert EEG data into images. In [31], the authors employed Wavelet Packet Decomposition (WPD) followed by a multiple CSP method to extract time and spatial features. These features were then used as inputs for an artificial neural network (ANN) model, resulting in an accuracy of 59.13%. The authors in [6] proposed a CNN and Riemannian Geometry Network (CRGNet) that was validated at an accuracy of 82.10% using k-fold cross-validation.

As shown in previous related papers, EEGNet has yielded promising results in EEG data classification. However, there is room for improvement in accuracy and further development. The structure should be enhanced to ensure an effective model for both binary and multiclass classification, as well as for various approaches, including within-subject and cross-subject strategies. Therefore, our goal is to provide a model that enhances accuracy across these conditions. We leverage the advantages of EEGNet by integrating it with a fusion technique and an attention block, enabling us to implement MI classification methods for both two and four MI tasks and for both within-subject and cross-subject strategies.

### 2.2. Channel Selection

An absence of a channel selection algorithm in BCI systems can result in noisy data and redundant channels, which in turn can lead to increased equipment and computational costs. For that reason, finding optimal channels has the potential to enhance or stabilize classification outcomes [3]. In order to find the optimal channels automatically, several approaches were used in the literature, including feature selection algorithms. In the feature selection process, the optimal subset of features is chosen after preprocessing and feature extraction to enhance classification performance. Similarly, channel selection involves identifying the most effective channels before feature extraction and classification to reduce computational demands while still ensuring robust outcomes in terms of classification accuracy [32]. Clearly, similar methodologies can be employed in both cases, where the objective is to find the best combination of elements that will improve the results.

Different methods for selecting channels have been used with the BCI IV 2a dataset, specifically when dealing with four-class classification. Researchers in [18,19,20,21,22] employ three main classification techniques: one-vs-one, one-vs-rest, and multiclass classification. In both one-vs-one and one-vs-rest, the means are derived from multiple binary classifications. One-vs-one considers every possible pair of two classes, whereas one-vs-rest trains classifiers for each class against the others. In contrast, multiclass classification trains a single classifier to distinguish between all classes at the same time. The authors in [19] employed the Firefly algorithm for channel selection, achieving a classification accuracy of 83.97% using the ML classifier as a regularized SVM with a one-to-one classification method. The application of the Firefly algorithm aimed at reducing the number of channels involved in calculating weighted scores for each channel near a candidate solution. Using both one-vs-one and one-vs-rest approaches, the researchers in [20] utilized various ML techniques to compute the means of several binary classifications. They demonstrated the advantages of incorporating feature extraction, feature selection, and MDA-SOGWO channel selection to enhance classification accuracy, elevating it from 67.04% to 80.82%. Additionally, the authors of [18,21] demonstrate the use of DL classification with the one-vs-rest strategy to validate channel selection methods. In their work [18], the researchers employed CSPs for optimal channel selection, followed by Fast Fourier Transform (FFT) transformation before training the DL model. They employed two specific models for their experiments: Stacked Sparse Autoencoder (SSAE) and Deep Belief Network built with stacked Restricted Boltzmann Machines (DBN-RBM). Initially, the accuracy for the SSAE model was 71.00%, and for the DBN-RBM model, it was 68.44%. As a result of channel selection, the accuracy of the SSAE model increased to 71.31%, while that of the DBN-RBM model increased to 68.63%. As detailed in [21], the authors developed a channel selection approach based on the standard deviation of wavelet coefficients across channels. They implemented CSPs using a one-vs-rest strategy and then utilized a CNN model for data classification, achieving an accuracy of 75.03%. In the multiclass classification strategy, as explored by authors in [22], channels are selected based on various metrics such as Euclidean distance, Riemannian distance, Kullback–Leibler, and Wasserstein distance divergence as criteria. Feature extraction was carried out using the one-vs-rest strategy with CSPs, coupled with an SVM for ML classification. This study’s findings indicate that maintaining a uniform number of channels across the selection process yields an accuracy of 75.57%, which is lower than the 77.82% achieved when selecting varying numbers of channels.

Genetic algorithms (GAs) are one of the approaches that have been used as a feature selection to optimize the weight of the classification [33,34,35]. Further, genetic algorithms are applied in order to select the best subset of channels that provide the highest level of accuracy [36,37,38,39]. For further explanation, the researchers in [37] used a GA to select 10 ECoG electrodes from a set of 64. They subsequently employed multi-layer perceptions (MLPs) for classification on the BCI Competition III dataset, increasing accuracy from 67% to 80% after selecting 10 electrodes. The authors in [36] utilized GAs with various EEG classification methods. Among these methods, the SVM performed the best, with accuracy increasing from 94.69% to 96.07% after the GA. In their study [39], the authors introduced two ML methodologies for categorizing right-hand and right-foot motor imagery into two distinct classes. They utilized the Rayleigh coefficient (RC) to extract features and employed the SVM and FDA for classification purposes. The authors used the GA, sequential forward search (SFS), and sequential backward search (SBS) to select channels, demonstrating the GA’s effectiveness in enhancing accuracy and delivering superior results. The GA resulted in an average accuracy of 88.2%, while without channel selection, it achieved 76.68%.

As a general observation, utilizing GAs demonstrates promising potential to improve accuracy, which aligns with our objective. According to the channel selection strategy, most previous works focus only on selecting different channels for each subject based on the best accuracy results achieved, whereas my method uses fixed optimal channels uniformly across all subjects. Moreover, when it comes to channel selection, most researchers either use ML to perform classification or DL models based on the mean of various binary classifications. Due to these factors, our work will utilize GAs with a variety of channels and fixed channels as well as DL models to train a single classifier among all classes.

## 3. Materials and Methods

The purpose of this section is to introduce the main methods used to construct the general framework of our study. It starts by providing an overview of the dataset used, followed by an explanation of our framework. Further details are provided regarding the framework components, including the DL classification approach, and the channel selection technique.

To provide a better understanding of the methodology, we will briefly describe the dataset used.

### 3.1. Description of BCI Competition IV 2a Dataset [40]

**Figure 2:**
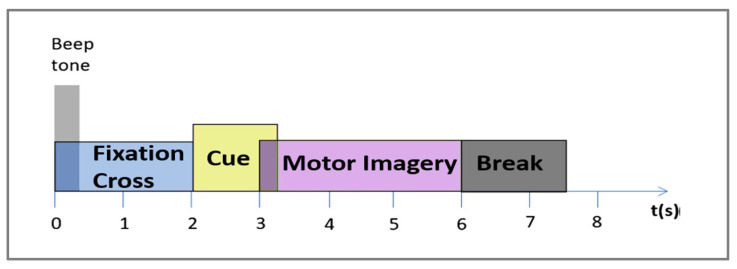
The timing scheme of the BCI IV 2a dataset.

This dataset consists of EEG data collected using 22 electrodes corresponding to the International 10–20 system from nine subjects. Four different motor imagery tasks were performed, including the imagination of the movement of the left hand (class 1), the right hand (class 2), both feet (class 3), and the tongue (class 4). There were two sessions recorded for each subject on two different days. A session consists of 288 trials divided into six runs, where one run contains 48 trials of the four possible classes. Samples were taken at 250 Hz, and bandpass filters were applied between 0.5 Hz and 100 Hz. As shown in Figure 2, the imagination period trial lasted for four seconds following cue onset and was terminated by the break at the end.

To explain the general structure of our work, we will explain the framework as a baseline for the rest of this unit.

### 3.2. Proposed Model Framework

**Figure 3:**
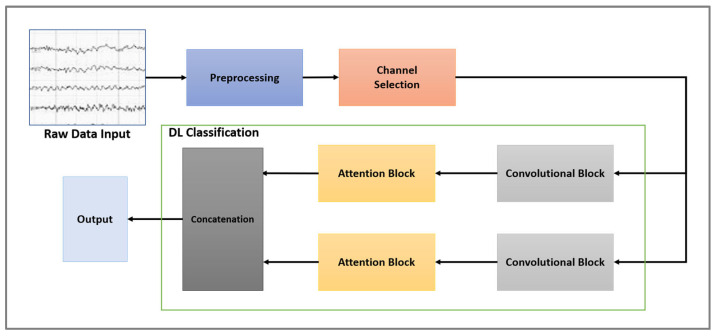
Overall framework.

Figure 3 provides an overview of the proposed framework, consisting of three primary stages handling raw EEG data: preprocessing, channel selection, and classification. EEG data input involves preprocessing as the first step in order to prepare the data for the purpose of distinguishing between MI tasks. The selected optimal channels are then forwarded for classification purposes. For classifying the input data, a two-level convolutional block followed by a CBAM attention block is applied. As a result, the output should indicate the correct MI task regardless of whether two or four classes are involved.

In the following sections, each stage is described in detail, beginning with preprocessing. We will then discuss the primary processes involved in classification. Finally, the method for selecting and determining the optimal channel will be clarified.

### 3.3. Preprocessing

In the preprocessing stage, we extracted windows of 4.5 s from each trial to better adapt to our classification needs [23,25]. As depicted in Figure 2, this included a four-second segment during the period of imagination and an additional half-second for the pre-cue onsets. With a sampling rate of 250 samples per second over a duration of 4.5 s, a total of 1125 samples were obtained.

The raw data were later filtered in the range of [0.25–50] using a 3rd order Butterworth filter as recommended in some previous research publications [41,42,43]. The filtering technique was selected for its effectiveness in removing frequencies that are not relevant to our study, particularly those below 0.25 Hz, which typically include slow drifts, and those above 50 Hz, mainly consisting of muscle noise and environmental electrical noise. The use of a bandpass filter ensures that the essential frequency components relevant to motor functions are retained, thereby enhancing the signal-to-noise ratio without distorting the underlying neural signatures. In addition, as demonstrated in Section 4.3.1, this filtering technique may enhance the quality of the EEG signal by eliminating certain frequencies of noise.

Our approach focused on preserving the raw signal characteristics; for this reason, no more complex preprocessing techniques were applied.

### 3.4. Classification

The main components of the FCNNA model will be discussed in this section, along with a breakdown of their structure. This model contains two layers of convolutional network blocks, followed by attention blocks. In the following paragraphs, we will take a closer look to give more details for each block:

#### 3.4.1. Convolutional Blocks

**Figure 4:**
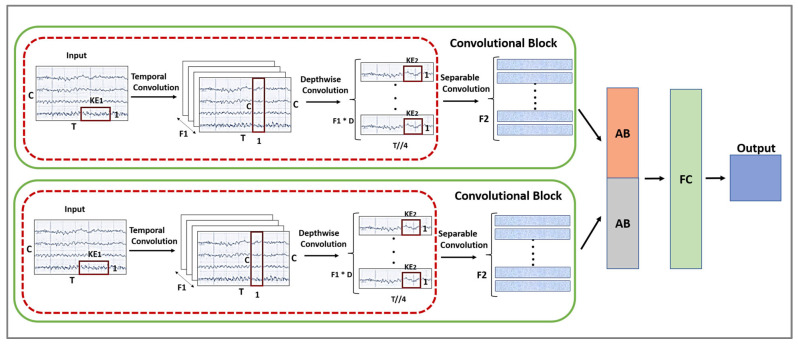
The structure of the FCNNA model.

The convolutional block is an enhanced version of EEGNet models using two layers with different hypermeters. By using two layers of the convolutional block, we will be able to obtain more accurate results, as explained later in the experiment section. Figure 4 illustrates the structure of the FCNNA model with a closer look at the convolutional block. The EEG data are taken as raw input to each convolutional block in the two presented layers. The output of each layer is an input of a separate attention block.

Each layer consists of two blocks: the block of the ConvNet structure and a separable convolutional block. The ConvNet block is described in Figure 1, where the frequency filter and spatial filter are applied to the EEG data in the same block. The frequency filter is applied on a specific time series of the raw data of each electrode separately; this time, the range depends on the kernel size. As part of our study, we applied the model to a 250 Hz EEG dataset, using 1×64  and 1×60  kernel sizes. Here, ‘1’ refers to the kernel height, indicating that each kernel processes one electrode channel at a time. Additionally, ‘64’ and ‘60’ represent the kernel width, corresponding to the number of consecutive temporal samples included in each convolution. This configuration enables us to examine and analyze EEG signals from each electrode, where the kernel widths process temporal segments approximately a quarter of a second long, based on our 250 Hz sampling rate. Later, a depthwise convolution is used to apply a spatial filter by learning from the features of all the electrodes in each frequency filter. After the depthwise convolution block, all electrodes become one as a result of frequency-specific spatial filters. The second block of each layer is a separable convolutional block, which is a combination of a depthwise convolution to apply a single frequency filter for each individual feature map, followed by a pointwise convolution. In a CNN, depthwise convolution and separable convolution provide efficient results since they reduce the number of parameters and computations [44].

Detailed information about the architecture of the convolutional block in the FCNNA model can be found in Table 1. This table shows the sequence of the model layers, the filter used, the kernel size of each convolutional block, and the shape of the output of each layer. The number of filters used is specified by three variables F1, D, and F2 where F1 is the temporal filter, D is the depth multiplier for the spatial filter, and when we multiply F1 by D, we obtain F2 which is the number of pointwise filters. It is important to note that we used different sizes of filters, kernels, and depths for each layer in the convolutional block. In the first layer, the kernel width of the first block is set to 60 temporal samples, and the number of temporal filters is 96 (denoted as F1) designed to extract different features from the input data. The layer has a depth of 2, meaning it doubles the total number of active filters to 192 (2 × F1) for depthwise convolution operations, enhancing feature extraction capabilities. On the other hand, the kernel width, depth, and filter F1 of the second layer are 64, 1, and 16, respectively. For both layers, we used the same second kernel size which is equal to 1×16. Furthermore, the table clarifies that batch normalization, activation, average pooling, and dropout are applied in sequence at the end of each block. Batch normalization is recommended to be used in the CNN model [45]. It standardizes the intermediate outputs of the network to zero mean and unit variance. This is meant to facilitate the optimization by keeping the inputs of layers closer to a normal distribution during training. Moreover, dropout randomly sets some inputs for a layer to zero in each training update to help prevent overfitting. The result later fits in the attention block (described in the next section) and is then merged for the classification.

#### 3.4.2. Attention Block

**Figure 5:**
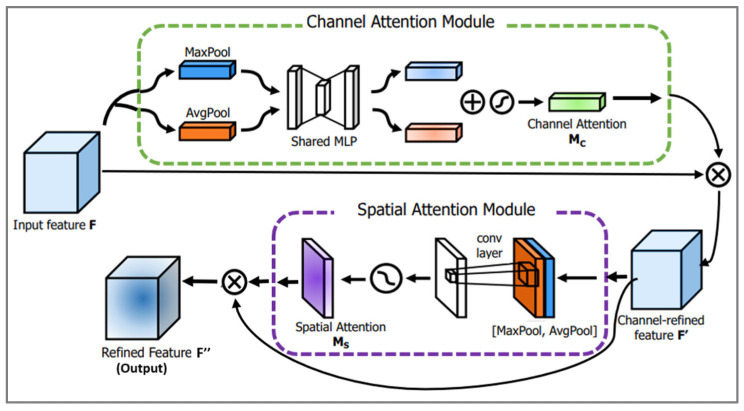
CBAM.

Attention mechanisms are DL techniques that allow the network to focus on different parts of its input. These mechanisms have been shown to be very effective in a variety of tasks, including image classification, natural language processing, and speech recognition [46,47,48]. One of these attention models is the Convolutional Block Attention Module (CBAM) [17]. The CBAM is often used together with other neural network models, such as CNNs, to improve their performance. It combines spatial and channel-specific attention mechanisms to improve the representation of features within input data. The two sub-module channels and spatial modules of the CBAM mechanism are illustrated in Figure 5. Starting with the channel attention module which receives the feature map F, $F\in R^{H\times W\times C}$, where in our mode, H = 1, W = 35, C = 192 for the first layer, and C = 16 for the second layer. This module returns Mc as defined in Equation (1), representing a 1D channel attention map that belongs to $R^{1\times 1\times C}$.

(1) $$Mc(F)=\sigma \left(MLP\left(AvgPool\left(F\right)\right)+MLP\left(MaxPool\left(F\right)\right)\right)$$

Mc(F) is calculated by subjecting the input feature maps F to both average and maximum pooling separately. These pooled results are then individually processed by three-layer feedforward artificial neural networks (MLP). The outcomes obtained from these MLPs are aggregated first and then processed through a sigmoid function (σ) to derive Mc. The result of Mc is then multiplied by the feature map F to give $F^{\prime }$, where $F^{\prime }$ is the context given by (3) and used by (4). On the other hand, spatial attention utilizes the input $F^{\prime }$ through a process involving average pooling and max pooling. This is followed by a convolution operation with a kernel size of 7, as shown in Equation (2).

(2) $$Ms(F^{\prime })=\sigma \left(f^{7\times 7}\left(\left[AvgPool\left(F^{\prime }\right);MaxPool\left(F^{\prime }\right)\right]\right)\right)$$

The main two formulas of the general process of this module are as below:

(3) $$F^{\prime }=Mc\left(F\right)⨂F$$

(4) $$F^{\prime \prime }=Ms\left(F^{\prime }\right)⨂F^{\prime }$$

where ⨂ denotes elementwise multiplication. Equation (4) illustrates the ultimate result of the CBAM. The refined feature, represented as $F^{\prime \prime }$, is obtained through the multiplication of Ms and $F^{\prime }$.

### 3.5. Channel Selection

This section will discuss the use of a genetic algorithm (GA) as a way of channel selection. In ML EEG classification, genetic algorithms are one of the most effective algorithms for channel selection [36,39]. The GA addresses a combinatorial optimization problem, aiming to select the optimal subset of EEG channels to maximize classification accuracy. This process involves evaluating various combinations of channels (chromosomes) and determining the most effective set based on a fitness function. This fitness function primarily measures classification accuracy, making it a crucial component in assessing the effectiveness of each channel combination. Genetic algorithms are particularly suited to this task as they can efficiently explore numerous potential combinations [36]. In our case, we applied GAs uniformly across all subjects to choose the optimal channels and determine the most appropriate combination that achieves the highest level of classification accuracy.

**Figure 6:**
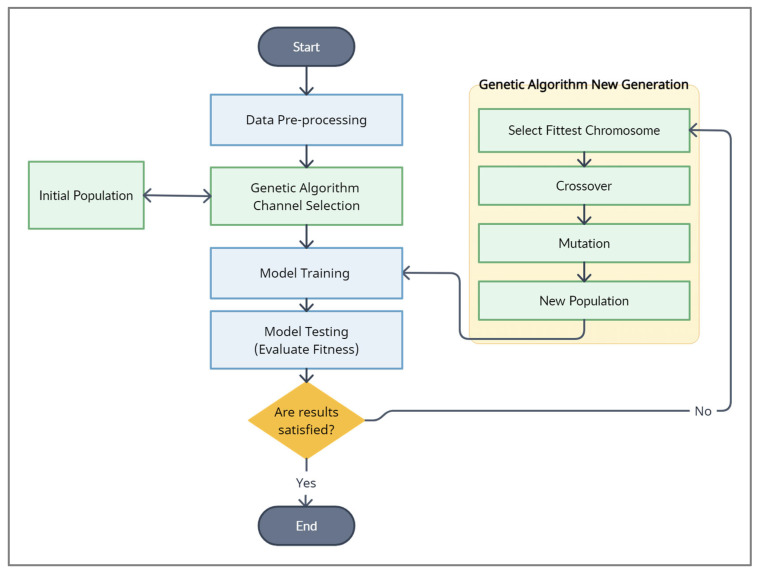
Genetic algorithm applied in our model.

The GA is a concept derived from science, where genes are the smallest components of a problem that eventually combine to form a chromosome. Specifically, the chromosome represents a possible solution to the problem. In our case, the gene is an input channel, and the chromosome is a combination of channels. In general, the algorithm consists of three main steps: First: Initialize the population by providing the initial possible solutions of a random set of channels. Second: Evaluate the fitness, which assesses each possible solution and validates the results. Third: Deliver a new generation based on choosing the solutions with the highest probability of achieving the greatest accuracy (parents) to generate a new generation of solutions (children). The new solution is generated after applying crossover and mutation. Figure 6 illustrates these steps and how they will be entered into our model. Here, in the following, we will explain each of these three steps in detail.

**Step1: Initialize the population:**

In this step, we will introduce a population consisting of n chromosomes, each carrying a random number of genes in order to ensure that every potential solution is possible. The population is denoted as {X1, X2, …, Xn}, where X represents a chromosome. For our study, we specifically set n to equal 6. The chromosome comprises a random selection of channels (genes) chosen from a list ranging from 1 to 22, representing the primary electrodes from the BCI Competition IV 2a dataset: [Fz, FC3, FC1, FCz, FC2, FC4, C5, C3, C1, Cz, C2, C4, C6, CP3, CP1, CPz, CP2, CP4, P1, Pz, P2, POz].

**Step 2: Evaluate the Fitness:**

Once the initial population has been prepared, each chromosome in the population is treated as a parent. Our classification process, as explained in Section 3.4.1, is utilized at this stage to assess each parent. Further, the dataset is divided into training and testing trials using a cross-subject strategy, details of which can be found in Section 4.1 and Section 4.4. Accordingly, a model is trained using only the channels included in that parent. Through this approach, the model’s performance reflects the effectiveness of the specific subset of channels. To evaluate the chosen channel subset, we examine the model using the test dataset and compute accuracy, denoted as $f\left(x\right).$

**Step 3: Deliver a new generation:**

If the classification results *f*(*x*) of the possible solution do not meet the threshold, a new generation will be provided. To deliver the new generation and have new children, these steps are followed:

- **Select the Fittest Chromosome:** The fitness-proportional roulette wheel approach is used to select three parents from a population. The mathematical formula for this approach can be found in Equation (5). In this approach, parents are selected based on their likelihood of having higher fitness values. From these parents, a new generation is produced.

(5) $$p=\frac{f(x)}{\sum_{j=0}^{n-1}f(x)}$$

- **Apply Crossover:** In order to perform crossover, two fitness parents are divided into halves, and then the genes are switched between them in the manner shown below.

$$Child\;1=\left[\left[Parent1\left[0\ldots p1\right]\right],\left[Parent2\left[p2\ldots end\right]\right]\right]$$

$$Child\;2=\left[\left[Parent2\left[0\ldots p3\right]\right],\left[Parent1\left[p4\ldots end\right]\right]\right]$$

where p1, p2, p3, p4 are the list index randomly chosen each time.

As an example, let us consider that we have these two fitness parents Parent1=[1,3,7,15,19,22] and Parent2=[2,7,15,21]. Assume that p1=3, p2=2, p3=2, and p4=4. As a result, the produced children are as follows:

$$Child\;1=\left[1,3,7,15,21\right]\;and\;Child\;2=[2,7,15,19,22]$$

- **Apply Mutation**

The mutation process was applied to the third parent with a probability of 0.5. This process entails altering certain genes within this parent; specifically, randomly chosen genes are modified to values not previously employed on this parent.

- **Generate a new population**

To complete the formation of the new population, we include the three newly generated offspring while eliminating three of the least fit chromosomes (parents) from the prior generation.

As a next step, we will repeat the algorithm, starting with Step 2, and continue until the threshold is met or the maximum generation time is reached.

## 4. Results and Discussion

In this section, we will examine our model’s classification performance using two different strategies. We intend to illustrate the differences in performance between our methodology and other state-of-the-art studies. Subsequently, we will demonstrate the performance after implementing channel selection.

### 4.1. Classification Strategy

For training and deriving the structure of our model, we used publicly available EEG data collected from the BCI Competition IV dataset. These data relate to four motor imaging tasks performed by a variety of subjects.

Training and Testing splitting:

To examine the BCI IV 2a dataset with our model, two techniques were applied for handling data from multiple subjects or individuals: within-subject and cross-subject. These strategies are used to determine how data are split, processed, and used for training and testing, with further elaboration provided in the subsequent sections.

Within-Subject strategy:

For BCI IV 2a, where each subject has two sessions, one of the sessions is used for training and the second session for testing. So, we have 288 trials in training and 288 trials in testing. Consequently, each subject will have a unique model that is used for individual classification.

Cross-subject strategy:

According to the idea of the cross-subject, one of the subjects is used for testing the model that was trained by the other subjects. In BCI IV 2a, we choose a different subject each time to test the model and combine its two sessions to obtain a total of 576 trials. Additionally, we included all 288 trials from both sessions of the remaining eight subjects, amounting to 4608 trials in total, to train this model.

Using these strategies, the dataset was classified into four motor imagery classes: the left hand, the right hand, both feet, and the tongue. Furthermore, we also classified two tasks based on left/right hands or feet/tongue.

### 4.2. Performance Metrics

Performance metrics are measurements used to evaluate the effectiveness of the proposed model. In our work, six different performance metrics are employed to assess specific aspects of the model which are accuracy, Kappa, precision, recall, the F1-score, and Receiver Operating Characteristic curves (ROC curves) [49]. As there are four distinct classes, the metrics are computed for each individual class. Nevertheless, in the case of accuracy, it is computed collectively across all classes to represent the overall classification accuracy for this particular model. In the following, you can see the definition and the equation of each metric [50,51].

Accuracy measures the proportion of correct predictions over the total predictions.

(6) $$Accuracy=\frac{TP+TN}{TP+FP+TN+FN}$$

Precision is a ratio of correct predictions for a specific class.

(7) $$Precision=\frac{TP}{TP+FP}$$

Recall measures how many of the positive classes are labeled correctly.

(8) $$Recall=\frac{TP}{TP+FN}$$

The F1-score is the harmonic mean or weighted average of precision and recall.

(9) $$F1\;score=2\times \frac{Precision\times Recall}{Precision+Recall}$$

In context, TP, TN, FP, and FN are defined as follows:

TP (True Positives) are the number of correct positive predictions.
TN (True Negatives) are the number of correct negative predictions.
FP (False Positives) are the number of incorrect positive predictions.
FN (False Negatives) are the number of incorrect negative predictions.

In the meantime, positive prediction refers to the class that the model identifies or predicts as the class of interest; on the other hand, negative prediction refers to the opposite class/classes.

The Cohen Kappa statistic (Kappa) is a metric that compares observed accuracy with expected accuracy (random chance). The model estimates how well it can classify instances correctly.

(10) $$Kappa=\frac{P_{o}-P_{e}}{1-P_{e}}$$

$P_{o}$ denotes the observed agreement which represents the proportion of times the classifiers agree on the classification of items. Moreover, $P_{e}$ refers to the expected agreement by chance which is derived based on the marginal probabilities of each classifier agreeing, considering only random chance.

Finally, ROC curves are visual representations of the binary classifier diagnostic ability as its discrimination threshold changes. These curves plot the true positive rate (sensitivity) on the y-axis versus the false positive rate (1—specificity) on the x-axis. The Area Under the ROC curve (AUC) ranges from 0.5 to 1.0, with values nearer 1.0 signifying higher authenticity and better classification performance. The AUC acts as a single numerical metric that encapsulates the ROC curve’s overall effectiveness, balancing sensitivity and specificity.

### 4.3. Classification Results

In our study, we built the FCNNA model using Python TensorFlow (version 2.15.0) and deployed it to the Google Colab platform equipped with a T4 GPU and 15.0 GB of GPU RAM. To ensure the robustness and reproducibility of our results, each model was trained ten times, with each session consisting of 1000 epochs. The models were trained using a batch size of 64 and a learning rate of 0.0009. A cross-entropy error function and an Adam optimizer were used to enhance learning efficiency. During the training phase, a callback function was used to save the model weights when the best accuracy was achieved, highlighting an efficient use of computational resources. The best model was then evaluated on the test set using comprehensive metrics, including accuracy, precision, recall, and F1-score. Additionally, we utilized ROC curves to assess the trade-offs between sensitivity and specificity and confusion matrices to provide a detailed breakdown of the model’s performance across different classes.

The following sections demonstrate the results of applying our model according to within-subject and cross-subject strategies.

#### 4.3.1. Within-Subject Classification

**Figure 7:**
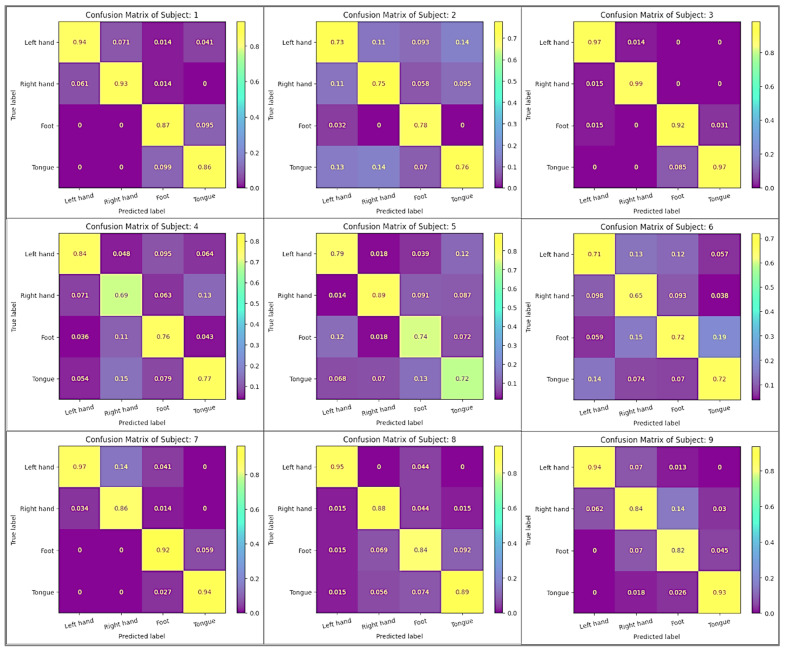
Confusion matrix of proposed model applied on 4 classes in BCI IV 2a using within-subject strategy.

By applying the suggested classification methodology to categorize the four tasks within the BCI IV 2a dataset for each subject separately (within-subject), the results obtained are presented in Table 2. Among the results obtained, a total of three subjects exceeded the 90% threshold, with Subject 3 particularly excelling with a 95.97% result. Furthermore, Subject 3 also demonstrated strong performance across all metric measurements. Figure 7 displays visual representations of the confusion matrix for each subject, which illustrates the difference in performance across classes on different subjects. According to the confusion matrix, the diagonal entries indicate how many predictions are accurate for each class. It can be concluded that higher values along this diagonal, from the top left to the bottom right, indicate more precise predictions for those particular classes.

In light of the use of a Butterworth filter in the preprocessing step of the classification process, Table 3 shows the results obtained with and without preprocessing. Despite the fact that Subjects 3 and 4 performed better without preprocessing, the general result is more accurate when preprocessing is performed. In Table 4, we illustrate the variations in accuracy when utilizing one, two, or three layers of the convolutional block in the classification process. It reveals that the two-layer configuration generally delivers the best performance, although it should be noted that Subjects 1 and 9 performed better with the three-layer configuration. Employing two layers offers an optimal balance by providing better accuracy while maintaining manageable complexity and processing time. This two-layer approach outperforms the single-layer configuration and avoids the increased computational demands associated with a three-layer configuration. Due to this, in our model, we chose to apply the preprocessing and a two-layer configuration of the convolutional block.

**Figure A1:**
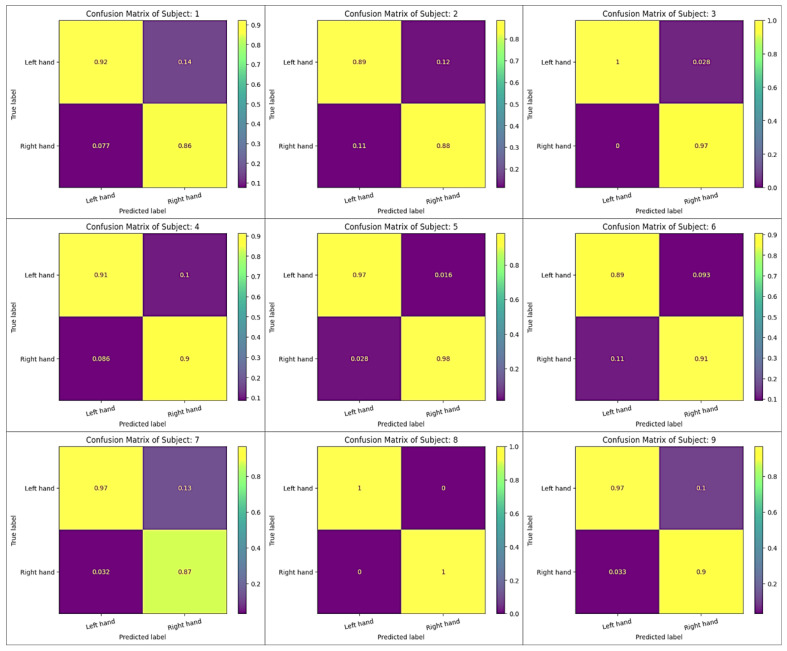
Confusion matrix of proposed model applied on 2 classes of left and right hand in BCI IV 2a using within-subject strategy.

**Figure A2:**
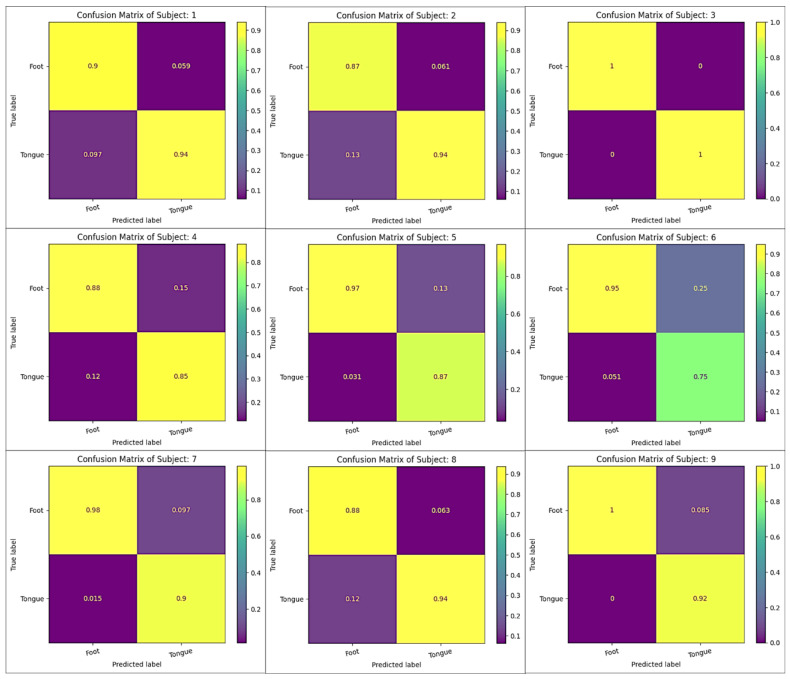
Confusion matrix of proposed model applied on 2 classes of both feet and tongue in BCI IV 2a using within-subject strategy.

We employed two validations of two classes to assess our model, as detailed in Table 5, demonstrating the outcomes for each combination. Specifically, we differentiated between the right hand and left hand, as well as between both feet and the tongue. The findings indicated that the average accuracy in classifying left and right hands yielded superior results of 93.09%. Notably, we achieved a 100% validation rate when classifying Subject 8 in class 1 and class 2, as well as Subject 3 in class 3 and class 4. Moreover, a confusion matrix of the model is shown in Figure A1 and Figure A2 of Appendix A to aid in the evaluation of the model’s performance when categorized into two classes, as well as identifying areas in which we may need to improve. Essentially, the confusion matrix visually presents how well the classifier performs by showcasing both correct and incorrect predictions for each class. The purpose of this visual aid is to enable you to evaluate the model’s precision, recall, accuracy, and overall performance.

As part of the comparison, we assess how well our model classifies four MI tasks compared with the baseline models [4] which are EEGNet [15], EEG-TCNet [23], MSFBCNN [24], and TCNet Fusion [25], ShallowNet [14], and DeepConvNet [14] as well as advanced models that are illustrated in Table 6 and Table 7. According to the results presented in Table 6, our study shows a significant improvement in the measured accuracy of 6.41% over most baseline models. In this regard, our results are comparable to those of study [25], in which the training phase was the same as in our study by using the callback function. Comparing the classification results of our model with advanced models in terms of accuracy, as shown in Table 7, reveals that the average performance of our study exceeded those of most other studies. As illustrated in Table 7, the highest accuracy values are noted in [4,26], and our model showcases remarkable consistency with variances of less than 1%. It is worth noting that studies [4,26] used 5-fold cross-validation to train their models. The authors in [4] merged all sessions and divided them into five groups. Each group was used as a validation set once, and the remaining four were utilized for training. Model accuracy is determined by averaging the maximum accuracy of the five folds. Meanwhile, the authors in [26] performed a random split, allocating 80% of each subject’s data for training and 20% for testing. Furthermore, our model significantly outperforms the models in [27,28,29] that incorporate EEGNet or ConvNet for performance enhancement. Additionally, Subject 2 and Subject 3 achieve the highest level of superiority in comparison with existing methods. Subject 2 experienced a 5% increase, while Subject 3 saw a 2% increase across previous advanced works. 

Table 8 compares the accuracy of our model with the state-of-the-art model using two classes of right-hand and left-hand classification. According to papers [8,52,53], ML techniques were employed to distinguish between two classes using Multi-task Transfer Learning (MTL), SVM, and LDA classifiers. In contrast, models based on DL were implemented in [28,54,55]. In particular, [28,54] utilized CNN architecture, while [55] utilized DBN and LSTM architecture. Based on the results, our model performed overwhelmingly well across most subjects and on average, as well.

#### 4.3.2. Cross-Subject Classification

As previously noted, the cross-subject strategy serves as a method to evaluate the model’s performance by assessing the data of each subject, which remain unutilized during the training phase. Table 9 shows the accuracy results of our model, where individual subject data are allocated for testing purposes. The results indicate that cross-subject strategies perform less well than within-subject strategies, which is reasonable considering that the data of the subjects in testing were not included in the training process. Despite this, the table also illustrates how we provide competitive results when compared with state-of-the-art works, with four subjects showing superior results. As indicated in the table, the EEGNet model [15], as well as EEG-TCNet [23], which includes a TCN block following EEGNet, and EEG Inception [56], which consists of two inception modules, demonstrates that our model performs better than those of others. According to [26,27], the authors assess their methodology based on two approaches: within-subject and cross-subject. Examining the data presented in Table 7 and Table 9 highlights the differences in performance between our model and the mentioned papers. In spite of the fact that [26] yields slightly better average results in a within-subject analysis, our model outperforms it when it comes to Subjects 1, 2, and 3. In contrast, our model produces better average results in cross-subject analysis and across all subjects, with the exception of Subject 9, where [26] performs slightly better. Moreover, our model outperforms all results presented in [27] in terms of within-subject accuracy. Although this study demonstrates notable outcomes in cross-subject analysis, Subject 3 in our model consistently maintains their superior performance, in both within-subject and cross-subject evaluations, along with Subjects 1, 6, and 8 in cross-subject analysis. It should be noted that the authors in [27] used the same splitting strategy as we did; however, they employed 10-fold cross-validation to divide the data into training and validation sets. For more detailed results of our model using cross-subject classification, refer to Table A1 of Appendix A, which includes metrics such as accuracy, Kappa, precision, recall, and the F1-score.

### 4.4. Channel Selection Results

In our study, we utilized a GA to select identical optimal channels for all subjects. The channels selected must maintain or increase the subjects’ performance. Consequently, employing a cross-subject strategy to evaluate the fitness ensured that the chosen channels were based on the most effective features identified by training and testing all subjects’ data simultaneously. Choosing channels can be a complex task since we need to apply the classification process several times to obtain the most accurate results. According to the GA, we applied three generations, where each generation has six populations to be evaluated. Therefore, 300 epochs were used to train and evaluate the results in a reasonable amount of time. Following this, we carried out three separate runs of the whole GA. After each run, the algorithm identified the top three sets of channels based on accuracy. We then selected the best-performing set of channels across all runs for use in our classification tasks.

**Figure 8:**
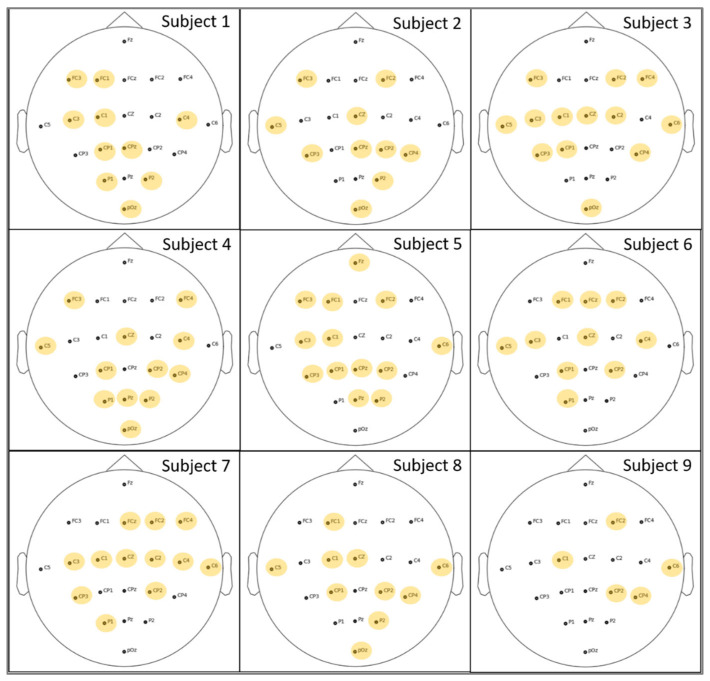
Optimal channels were selected based on GA and cross-subject classification after testing each subject individually. The highlighted electrodes indicate the positions of the selected channels for each subject.

Table 10 presents the selected channels which are chosen based on the best accuracy obtained after applying cross-subject classification through the GA to test each subject individually. Further clarification can be found in Figure 8, which illustrates the distribution of these optimal channels, which were selected from 22 main electrodes following the International 10–20 system.

**Figure 9:**
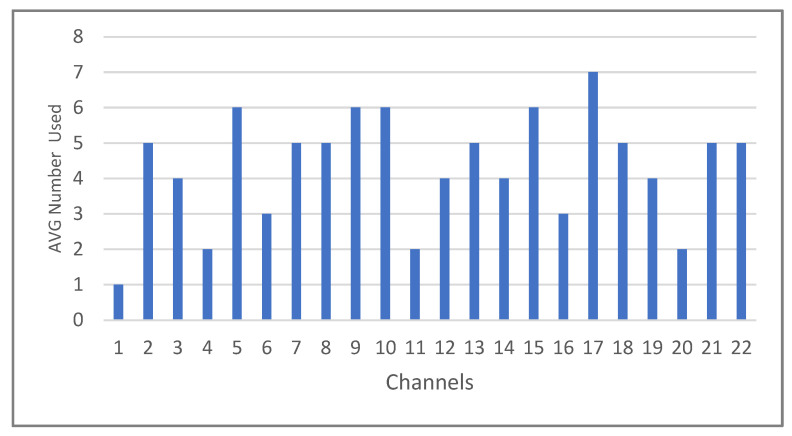
The average number of channels (electrodes) selected for all subjects after applying the genetic algorithm through cross-subject classification.

While no identical electrodes were selected, the results indicate that some electrodes were used more frequently than others. Figure 9 shows the average number of electrodes chosen for all subjects according to the different classification processes applied in Table 10. The most popular choice, channel 17, was chosen by seven out of nine participants. Following closely are channels 5, 9, and 10, which were all used by six subjects. In contrast, channel 1 was the least favored, chosen by only one subject, specifically Subject 5.

Cross-subject classification is used to validate the performance of the selected subset channels. Therefore, all subjects’ data were involved in the training or testing process. This will assist in the establishment of fixed optimal channels for all subjects. However, our goal is to choose one set of identical channels for all subjects. According to Table 10, testing Subject 3 provides the highest level of accuracy. Furthermore, by comparing the results in Table 10 (with channel selection) with Table 9 (using the entire electrodes), it is evident that testing Subject 1 shows a significant improvement of more than 4% when certain channels are reduced. As a result, it may appear that either the set of channels of Subject 1 or Subject 3 is the best for fixed channels. To verify this result, we evaluated the performance of each set of channels in Table 10 in order to choose the best combinations. The performance of the selected channels was measured based on within-subject classification using these selected channels. As expected, the highest average accuracy values are observed in channel sets associated with testing Subjects 1 and 3. For additional details, see Table A2 of Appendix A. Table 11 presents a detailed breakdown of the accuracy, Kappa values, and time duration for the within-subject classification results after applying the selected channels obtained from testing Subject 1 and Subject 3. Additionally, the results of a full channel classification are presented so that a clear comparison can be made. The results indicate that channel selections improve the performance reflected by the used metrics for many subjects, including Subject 1, Subject 2, Subject 4, Subject 6, and Subject 8. Specifically, Subject 6 exhibits increased accuracy with both combinations of channel selection. In addition, the classification duration is reduced from one and a half hours to two hours, resulting in more efficient results. Therefore, we will consider the set of channels “[2, 3, 8, 9, 12, 15, 16, 19, 21, 22]” that are produced by testing Subject 1 as the fixed optimal in our proposed work since they provide the best accuracy with the shortest computation time.

Selecting the optimal channels for each subject contributes to reducing noises and increasing the accuracy. In depth, after using within-subject classification across the channel sets listed in Table 10, we identified channel sets that improved accuracy for certain subjects. Table 12 demonstrates variable optimal channels for each subject, presenting their corresponding accuracy results. The table represents the cross-subject experiments conducted to determine these optimal channels. Furthermore, it shows a significant increase in overall accuracy as well as substantial improvements in individual accuracy.

**Figure 10:**
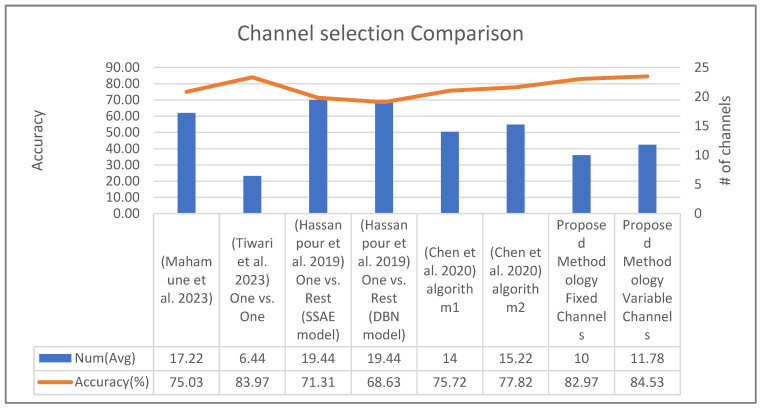
A comparison of our work with the state-of-the-art research in terms of accuracy and the number of channels used, as discussed in Hassanpour et al. (2019) [18], Tiwari et al. (2023) [19], Mahamune et al. (2023) [21], and Chen et al. (2020) [22].

Below is a comparison of our proposed work with the existing state-of-the-art related research contributions according to the channel selection process. As shown in Table 13, our study provides an advantage over the previous work in terms of the identicality of the channels and used strategy. In particular, the proposed variable channel approach differs from previous studies in the fact that it combines two strategies, resulting in the highest accuracy. As well as using a new strategy, our fixed channel approach also uses the same channels for all subjects, resulting in a significant increase in accuracy. Figure 10 demonstrates how our work performs compared to the previous one by presenting the number of channels used and the average accuracy obtained through the channel selection process. As shown by the figure, the proposed variable channel methodology demonstrates the highest degree of accuracy, followed by the work presented in [19], and then our fixed channel methodology. While the study in [19] achieves notable accuracy with fewer channels, it employs the one-vs-one strategy, which involves the use of multiple binary classifications and averages their results. In contrast, our approach uses a single classifier to accurately differentiate between four classes, achieving not only higher accuracy but also a more efficient reduction in the number of channels used.

Finally, in our experiment, we validated classification performance based on four distinct methods: within-subject with all channels, cross-subject, within-subject with fixed channels, and within-subject with variable channels. We found that the within-subject method utilizing variable channels achieved the most significant results within a reasonable duration. Conversely, the within-subject strategy with a fixed set of channels produced the highest accuracy and Kappa values, particularly when considering the shortest computation time. Standardizing the number of channels across all participants has proven to be a particularly effective technique. This technique produces impressive results that demonstrate the method’s effectiveness and importance in terms of accuracy, Kappa scores, and processing time. This novel approach represents a significant advancement in the field, potentially introducing an innovative direction in how channels could be selected. It allows for the use of a consistent set of channels across all subjects, a method not previously applied. This approach not only challenges traditional approaches but also addresses existing channel selection limitations, opening up new opportunities for research and application.

Based on our results, we can say that all four methods demonstrated high accuracy in classification, overcoming the average accuracy value shown in most similar studies. The within-subject with all channels method was assessed with two-class and four-class classifications. The two-class classification achieved the highest accuracy among all studies, while the four-class classification recorded the highest accuracy in most studies and exceeded all others for Subjects 2 and 3. Subjects 1, 3, 6, and 8 achieved the highest accuracy scores using cross-subject classification, delivering competitive results on average. The within-subject with variable channel method outperformed previous studies in accuracy by using an average of 11.78 channels. On the other hand, the within-subject with fixed channels method was more accurate than most other studies, except for one. By employing a single classifier, our method distinguishes itself from this previous study, which used a one-to-one approach.

**Figure 11:**
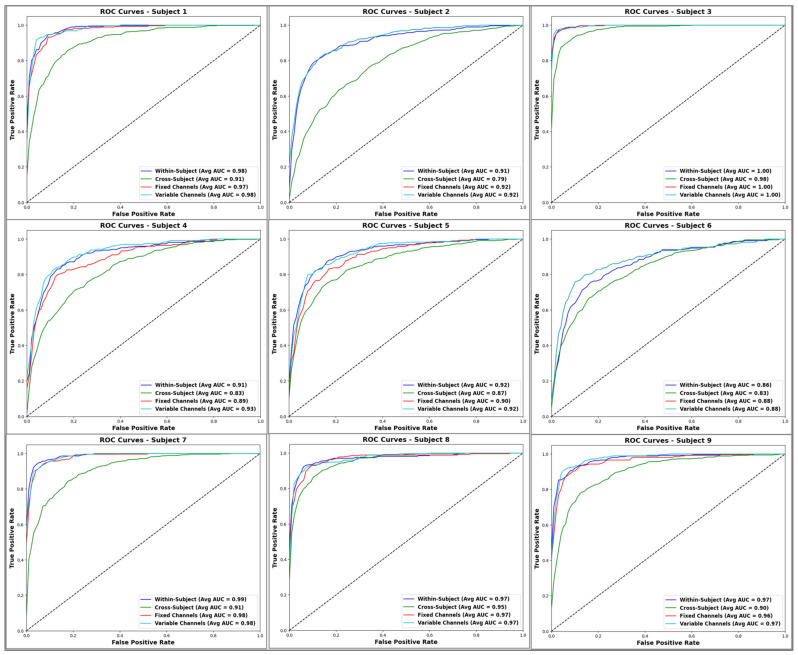
ROC curves and AUC for each subject across different methods: within-subject with all channels (“Within-subject”), cross-subject, within-subject with fixed channel selection (“Fixed Channels”), and within-subject with variable channel selection (“Variable Channels”). The dotted black lines reflect the performance of a random predictor, serving as a reference for comparing the classification performance of the four methods.

For further evaluation, Figure 11 illustrates the ROC curve and AUC for each subject across these four methods. The results of the AUC are generally good, with most scores remaining above 90. There is no doubt that the AUC for Subject 3 is impressive, scoring 100 for the within-subject method, 98 for the cross-subject method, and 100 for both the fixed and variable channel methods. Contrary to this, the cross-subject method yields the lowest AUC, particularly for Subject 2, where it reaches 79. Additionally, all the within-subject methods, either with all channels or with selected channels, demonstrate similar results, showing the various channel selection strategies consistently outperforming the others.

## 5. Conclusions

In the research area addressing MI-EEG-based BCIs, several challenges limit the growth of classification accuracy involving the complexity and the redundancy of EEG signal data. In this paper, we presented a Fusion convolutional neural network with Attention blocks (FCNNA) model to perform multiclass classification with a channel selection mechanism. Our approach began with preprocessing to eliminate noise and prepare the EEG raw data. Afterward, the FCNNA model was used for classification, which consists of layers of convolutional blocks followed by a CBAM attention block. Based on a comparison between one, two, and three layers of convolutional blocks, it was determined that two layers provide the best performance in terms of accuracy, complexity, and processing time. Lastly, a genetic algorithm was used for channel selection. The novelty of this stage is the use of a new technique that combines cross-subject and within-subject methods. Many cross-subject classifications were applied through the channel selection process to provide various sets of optimal channels. Following this, within-subject classifications were performed so that fixed and variable channels can be selected for each subject.

The experimental results on BCI IV 2a showed that our method effectively addressed the issues of existing CNN-based EEG motor imagery classification and improved the performance. Our proposed work is evaluated through four different scenarios: within-subject classification, cross-subject classification, a fixed set of channel selection, and a variable set of channel selection. As a result of our within-subject strategy, multiclass classification showed an impressive improvement of 83.78%. The accuracy of the model was considered to be higher than the EEGNet, MSFBCNN, EEG-TCNet, ShallowNet, and DeepConvNet models by 6.41%. Moreover, in comparison to advanced works, Subject 2 had a 5% increase in accuracy, and Subject 3 had a 2% increase. In addition, a within-subject strategy with two classes resulted in the best performance at 93.09%. The second multiclass classification applied using a cross-subject strategy resulted in an impressive accuracy score of 68.87%. In both scenarios, the fixed set of channels and the variable set of channels, only one classifier was used to distinguish between the four classes with a superior accuracy of 82.97% and 84.53%, with an average number of channels between 10 and 11.78. As a result of analyzing the four scenarios, the within-subject method employing variable channels achieved the highest accuracy and Kappa results. Meanwhile, the strategy with a fixed set of channels achieved the highest accuracy in the shortest computation time.

In future work, we intend to improve performance and efficiency through the incorporation of transfer learning. Using the concepts introduced in this paper, we aim to further develop classification methods and channel selection techniques to improve performance. This will significantly contribute to the advancement of BCI systems. Additionally, the insights gained from our study collectively suggest promising directions for future research and practical applications in EEG MI classification and EEG channel selection.

## Figures and Tables

**Table 1:** A detailed description of the convolutional block in the FCNNA model, where C = number of channels (22), T = number of time points (1125), F1 = number of temporal filters [ F1First = 96, F1Second = 16], D = depth multiplier (number of spatial filters) [DFirst = 2, DSecond = 1], F2 = number of pointwise filters [F2 = F1 × D], and N = number of classes, KE = kernel width [KE1First = 60, KE1Second = 64, KE2 = 16].

Block	Layer	Layer Type	# of Filter	Kernel Size	Output	Option
First Convolutional Block						
1	Input	Input			(C, T)	
	Reshape				(C, T, 1)	
	C1	Conv2D	F1	(1, KE1)	(C, T, F1)	padding = ‘same’
	BN1	BatchNorm			(C, T, F1)	
	C2	Depthwise Conv2	F1 × D	(C, 1)	(1, T, F1 × D)	depth_multiplier = D, max norm = 1
	BN2	BatchNorm			(1, T, F1 × D)	
	A1	Activation			(1, T, F1 × D)	Function = ELU
	P1	AveragePooling2D		(1, 4)	(1, T//4, F1 × D)	
	D1	Dropout				*p* = 0.5
2	C3	Separable Conv2	F2	(1, KE2)	(1, T//4, F2)	
	BN3	BatchNorm			(1, T//4, F2)	
	A2	Activation			(1, T//4, F2)	Function = ELU
	P2	AveragePooling2D		(1, 8)	(1, T//32, F2)	
	D2	Dropout			(1, T//32, F2)	*p* = 0.5
	AB	Attention Block			(1, T//32, F2first)	
Second Convolutional Block “the same Previous Step”						
	AB	Attention Block			(1, T//32, F2Second)	
	Con1	Concatenation			(1, T//32, F2first+ F2Second)	
	FC1	Flatten			(T//32 × (F2first+ F2Second))	
	Dense	Dense			N	Softmax

**Table 2:** The results of our model using the within-subject strategy of 4 classes in the BCI IV 2a dataset.

	Accuracy	Kappa	Precision	Recall	F1-Score
Subject 1	90.04%	86.71%	90%	90%	90%
Subject 2	75.62%	67.51%	75%	76%	75%
Subject 3	95.97%	94.63%	96%	96%	96%
Subject 4	76.32%	68.38%	77%	76%	76%
Subject 5	78.26%	70.98%	79%	78%	78%
Subject 6	69.30%	59.05%	70%	69%	69%
Subject 7	91.70%	88.93%	92%	92%	92%
Subject 8	88.93%	85.24%	89%	89%	89%
Subject 9	87.88%	83.83%	88%	88%	88%
AVG	83.78%	78.36%			

**Table 3:** A comparison of the classification accuracy between the preprocessed and unprocessed BCI IV 2a dataset using within-subject four-class analysis. The best scores are shown in bold.

	Sub 1	Sub 2	Sub 3	Sub 4	Sub 5	Sub 6	Sub 7	Sub 8	Sub 9	AVG
No Preprocessing Accuracy	87.54%	72.08%	**97.07%**	**80.26%**	77.54%	68.84%	90.97%	88.19%	87.50%	83.33%
With Preprocessing Accuracy	**90.04%**	**75.62%**	95.97%	76.32%	**78.26%**	**69.30%**	**91.70%**	**88.93%**	**87.88%**	**83.78%**

**Table 4:** A comparison of the classification accuracy between one-layer, two-layer, and three-layer convolutional blocks in the BCI IV 2a dataset using four within-subject classes. The best scores are shown in bold.

	Sub 1	Sub 2	Sub 3	Sub 4	Sub 5	Sub 6	Sub 7	Sub 8	Sub 9	AVG
One-Layer	89.32%	74.20%	95.24%	69.74%	75.72%	60.47%	90.97%	85.98%	87.50%	81.02%
Two-Layer	90.04%	**75.62%**	**95.97%**	**76.32%**	**78.26%**	**69.30%**	**91.70%**	**88.93%**	87.88%	**83.78%**
Three-Layer	**90.75%**	74.56%	95.60%	74.56%	77.54%	68.37%	90.61%	88.56%	**88.64%**	83.24%

**Table 5:** The within-subject classification accuracy of two classes in the BCI IV 2a dataset, classified between class 1 (left hand) and class 2 (right hand) and also between class 3 (both feet) and class 4 (tongue).

	Sub 1	Sub 2	Sub 3	Sub 4	Sub 5	Sub 6	Sub 7	Sub 8	Sub 9	AVG
Class 1 and Class 2	88.65%	88.03%	98.54%	90.52%	97.78%	89.81%	91.43%	100.00%	93.08%	93.09%
Class 3 and Class 4	92.14%	90.07%	100.00%	86.61%	91.49%	82.24%	94.16%	90.51%	95.52%	91.42%

**Table 6:** A comparison of our model accuracy with the baseline model on the BCI IV 2a dataset using a within-subject approach. The best scores are shown in bold.

	ShallowNet [4]	DeepConvNet [4]	EEGNet [4]	MSFBCNN [4]	EEG-TCNet [23]	TCNet Fusion [25]	Our Model
Accuracy	74.31%	75.64%	73.39%	75.12%	77.35%	83.73%	**83.78%**
Kappa	0.66	0.67	0.65	0.67	0.70	0.78	0.78

**Table 7:** A comparison of our model accuracy with the state-of-the-art model on the BCI IV 2a dataset using a within-subject approach. The best scores are shown in bold.

	CMO-CNN [26]	SSSTN [30]	SDDA [29]	MTFB-CNN [4]	CRGNet [6]	EEG-ITNet [27]	Echtioui et al. [31]	MBSTCNN-ECA-LightGBM [28]	Our Model
Subject 1	86.95%	86.46%	**90.62%**	90.52%	83.80%	84.38%	70.1 7%	82%	90.04%
Subject 2	67.47%	58.33%	62.84%	68.10%	70.60%	62.85%	57.75%	61%	**75.62%**
Subject 3	92.69%	92.57%	93.40%	93.97%	90.80%	89.93%	83.62%	89%	**95.97%**
Subject 4	77.21%	75.35%	**84.02%**	74.14%	75.60%	69.10%	38.79%	63%	76.32%
Subject 5	**82.78%**	80.90%	68.05%	80.17%	80.60%	74.31%	47.41%	71%	78.26%
Subject 6	**73.73%**	67.01%	61.80%	72.41%	73.00%	57.64%	38.96%	64%	69.30%
Subject 7	92.52%	93.06%	**97.20%**	96.55%	95.80%	88.54%	74.82%	72%	91.70%
Subject 8	90.43%	85.76%	90.97%	**91.38%**	89.20%	83.68%	69.65%	79%	88.93%
Subject 9	91.47%	86.46%	89.23%	**93.10%**	79.30%	80.21%	51.03%	84%	87.88%
AVG	83.92%	80.66%	82.01%	**84.48%**	82.10%	76.74%	59.13%	74%	83.78%

**Table 8:** A comparison of the accuracy of two classes (left hand and right) between our model and the current state-of-the-art models on the BCI IV 2a dataset using a within-subject approach. The best scores are shown in bold.

	HS-CNN [54]	SW-LCR [8]	CSP+ DBN [55]	CSP+ LSTM [55]	KMDA [53]	VFB-RCSP [52]	MBSTCNN-ECA-LightGBM [28]	Our Model
Subject 1	**90.07%**	86.81%	48.15%	48.15%	79.01%	86.11%	88%	88.65%
Subject 2	80.28%	64.58%	51.85%	51.85%	72.52%	70.83%	78%	**88.03%**
Subject 3	97.08%	95.83%	48.15%	51.85%	90.25%	94.44%	87%	**98.54%**
Subject 4	89.66%	67.36%	50.00%	50.00%	70.25%	73.61%	76%	**90.52%**
Subject 5	97.04%	68.06%	50.00%	50.00%	68.55%	61.11%	93%	**97.78%**
Subject 6	87.04%	67.36%	52.38%	47.62%	71.02%	70.83%	77%	**89.81%**
Subject 7	**92.14%**	80.56%	50.00%	50.00%	88.29%	63.89%	87%	91.43%
Subject 8	98.51%	97.22%	50.00%	50.00%	90.71%	93.06%	94%	**100.00%**
Subject 9	92.31%	92.36%	52.63%	47.37%	90.66%	88.19%	78%	**93.08%**
AVG	91.57%	80.02%	50.35%	49.65%	80.14%	78.01%	84%	**93.09%**

**Table 9:** A comparison of our model accuracy with the state-of-the-art model on the BCI IV 2a dataset using a cross-subject approach. The best scores are shown in bold.

	CMO-CNN [26]	EEG-ITNet [27]	EEG Inception [27]	EEGNet 8,2 [27]	EEG-TCNet [27]	Our Model
Subject 1	68.75%	71.88%	66.32%	68.75%	69.10%	**72.56%**
Subject 2	44.44%	**62.85%**	48.26%	50.00%	52.08%	53.71%
Subject 3	78.47%	81.94%	73.61%	80.21%	81.94%	**86.37%**
Subject 4	55.90%	**65.62%**	56.60%	59.38%	61.81%	60.82%
Subject 5	53.12%	63.19%	**65.62%**	64.24%	60.42%	59.11%
Subject 6	51.56%	56.25%	56.25%	48.26%	51.39%	**59.91%**
Subject 7	67.70%	**80.21%**	73.61%	72.57%	76.39%	72.63%
Subject 8	76.38%	78.12%	70.49%	77.43%	74.31%	**81.68%**
Subject 9	**73.78%**	64.93%	61.11%	55.56%	58.68%	73.05%
AVG	63.34%	**69.44%**	63.54%	64.04%	65.12%	68.87%

**Table 10:** Chosen channels based on best accuracy results using genetic algorithm and cross-subject classification.

	Selected Channels	# of Channels	Accuracy
Subject 1 in Testing	[2, 3, 8, 9, 12, 15, 16, 19, 21, 22]	10	76.90%
Subject 2 in Testing	[2, 5, 7, 10, 14, 16, 17, 18, 21, 22]	10	52.98%
Subject 3 in Testing	[2, 5, 6, 7, 8, 9, 10, 11, 13, 14, 15, 18, 22]	13	85.82%
Subject 4 in Testing	[2, 6, 7, 10, 12, 15, 17, 18, 19, 20, 21, 22]	12	61.22%
Subject 5 in Testing	[1, 2, 3, 5, 8, 9, 13, 14, 15, 16, 17, 20, 21]	13	57.81%
Subject 6 in Testing	[3, 4, 5, 7, 8, 10, 12, 15, 17, 19]	10	58.06%
Subject 7 in Testing	[4, 5, 6, 8, 9, 10, 11, 12, 13, 14, 17, 19]	12	68.25%
Subject 8 in Testing	[3, 7, 9, 10, 13, 15, 17, 18, 21, 22]	10	80.56%
Subject 9 in Testing	[5, 9, 13, 17, 18]	5	70.66%

**Table 11:** Within-subject classification based on the fixed channel selections. The best scores are shown in bold.

	Optimal Channels of Subject 1 in Testing [2, 3, 8, 9, 12, 15, 16, 19, 21, 22]		Optimal Channels of Subject 3 in Testing [2, 5, 6, 7, 8, 9, 10, 11, 13, 14, 15, 18, 22]		All Channels Classification [1,2, 3, 4, 5, 6, 7, 8, 9, 10, 11, 12, 13, 14, 15, 16, 17, 18, 19, 20, 21, 22]	
	**Accuracy**	**Kappa**	**Accuracy**	**Kappa**	**Accuracy**	**Kappa**
Subject 1	87.90%	83.86%	**90.75%**	87.67%	90.04%	86.71%
Subject 2	**77.39%**	69.86%	67.84%	57.16%	75.62%	67.51%
Subject 3	95.60%	94.14%	94.51%	92.67%	**95.97%**	94.63%
Subject 4	75.88%	67.77%	**77.19%**	69.50%	76.32%	68.38%
Subject 5	73.19%	64.30%	77.90%	70.51%	**78.26%**	70.98%
Subject 6	**73.02%**	64.03%	**70.23%**	60.29%	69.30%	59.05%
Subject 7	88.81%	85.09%	89.53%	86.05%	**91.70%**	88.93%
Subject 8	87.82%	83.76%	**89.67%**	86.22%	88.93%	85.24%
Subject 9	87.12%	82.81%	84.85%	79.77%	**87.88%**	83.83%
AVG	82.97%		82.50%		83.78%	78.36%
Time Duration	2 h: 36 min		3 h: 04 min		4 h: 38 min	

**Table 12:** Variable optimal channels for each subject.

	Accuracy	Kappa	Variable Optimal Channels	Belong to Which Cross-Subject	Time Duration
Subject 1	90.75%	87.67%	[2, 5, 6, 7, 8, 9, 10, 11, 13, 14, 15, 18, 22]	Testing Subject 3	3:04 h
Subject 2	77.39%	69.86%	[2, 3, 8, 9, 12, 15, 16, 19, 21, 22]	Testing Subject 1	2:36 h
Subject 3	96.34%	95.12%	[2, 5, 7, 10, 14, 16, 17, 18, 21, 22]	Testing Subject 2	2:34 h
Subject 4	77.19%	69.50%	[2, 5, 6, 7, 8, 9, 10, 11, 13, 14, 15, 18, 22]	Testing Subject 3	3:04 h
Subject 5	77.90%	70.51%	[2, 5, 6, 7, 8, 9, 10, 11, 13, 14, 15, 18, 22]	Testing Subject 3	3:04 h
Subject 6	73.02%	64.03%	[2, 3, 8, 9, 12, 15, 16, 19, 21, 22]	Testing Subject 1	2:36 h
Subject 7	89.53%	86.05%	[2, 5, 6, 7, 8, 9, 10, 11, 13, 14, 15, 18, 22]	Testing Subject 3	3:04 h
Subject 8	89.67%	86.22%	[2, 6, 7, 10, 12, 15, 17, 18, 19, 20, 21, 22]	Testing Subject 4	2:51 h
Subject 9	89.02%	89.02%	[2, 6, 7, 10, 12, 15, 17, 18, 19, 20, 21, 22]	Testing Subject 4	2:51 h
AVG	84.53%	79.78%			2:51 h

**Table 13:** A comparison between our work and the state-of-the-art research on channel selection in the BCI IV 2a dataset.

	Accuracy	#of Channels	Use the Same Number of Channels	Use the Same Channels?	Strategy
(Mahamune et al. 2023) [21]	75.03%	17.22	No	No	Within-subject
(Tiwari et al. 2023) [19]	83.97%	6.44	No	No	One vs. One
(Hassanpour et al. 2019) [18] (SSAE model)	71.31%	19.44	No	No	One vs. Rest
(Hassanpour et al. 2019) [18] (DBN model)	68.63%	19.44	No	No	One vs. Rest
(Chen et al. 2020) [22] algorithm1	75.72%	14	Yes	No	Within-subject in classification One vs. Rest in feature extraction
(Chen et al. 2020) [22] algorithm2	77.82%	15.22	No	No	Within-subject in classification One vs. Rest in feature extraction
Proposed Methodology Fixed Channels	82.97%	10	Yes	Yes	Cross-subject in channel selection Within-subject in classification
Proposed Methodology Variable Channels	84.53%	11.78	No	No	Cross-subject in channel selection Within-subject in classification

## Data Availability

The BCI-IV-2a dataset can be downloaded from the following link: https://www.bbci.de/competition/iv/#datasets, accessed on 10 March 2023.

## Appendix A

**Table A1:** Cross-subject classification results of 4 classes in the BCI IV 2a dataset.

	Accuracy	Kappa	Precision	Recall	F1-Score
Subject 1	72.56%	63.42%	74%	73%	73%
Subject 2	53.71%	38.29%	55%	54%	54%
Subject 3	86.37%	81.83%	87%	86%	86%
Subject 4	60.82%	47.63%	65%	61%	61%
Subject 5	59.11%	45.62%	62%	59%	58%
Subject 6	59.91%	46.52%	61%	60%	59%
Subject 7	72.63%	63.52%	74%	73%	73%
Subject 8	81.68%	75.57%	82%	82%	82%
Subject 9	73.05%	64.06%	73%	73%	73%
AVG	68.87%	58.50%			

**Table A2:** Within-subject classification based on the set of channels in Table 10.

	Table 10 Selected Channels According to								
	Testing Subject 1	Testing Subject 2	Testing Subject 3	Testing Subject 4	Testing Subject 5	Testing Subject 6	Testing Subject 7	Testing Subject 8	Testing Subject 9
Accuracy (Avg)	82.97%	81.69%	82.5%	81.54%	79.87%	80.49%	80.05%	79.94%	69.46%

## References

[1] Ramadan R.A., Refat S., Elshahed M.A., Rasha A.A., Hassanien A.E., Azar A.T. (2015). Basics of Brain Computer Interface. Brain-Computer Interfaces. Intelligent Systems Reference Library.

[2] Pfurtscheller G., Neuper C. (2001). Motor Imagery and Direct Brain-Computer Communication. Proc. IEEE.

[3] Baig M.Z., Aslam N., Shum H.P.H. (2020). Filtering Techniques for Channel Selection in Motor Imagery EEG Applications: A Survey. Artif. Intell. Rev..

[4] Li H., Chen H., Jia Z., Zhang R., Yin F. (2023). A Parallel Multi-Scale Time-Frequency Block Convolutional Neural Network Based on Channel Attention Module for Motor Imagery Classification. Biomed. Signal Process. Control.

[5] Varsehi H., Firoozabadi S.M.P. (2021). An EEG Channel Selection Method for Motor Imagery Based Brain–Computer Interface and Neurofeedback Using Granger Causality. Neural Netw..

[6] Gao C., Liu W., Yang X. (2022). Convolutional Neural Network and Riemannian Geometry Hybrid Approach for Motor Imagery Classification. Neurocomputing.

[7] Zancanaro A., Cisotto G., Paulo J.R., Pires G., Nunes U.J. (2021). CNN-Based Approaches For Cross-Subject Classification in Motor Imagery: From the State-of-the-Art to DynamicNet. Proceedings of the 2021 IEEE Conference on Computational Intelligence in Bioinformatics and Computational Biology (CIBCB).

[8] Gaur P., Gupta H., Chowdhury A., McCreadie K., Pachori R.B., Wang H. (2021). A Sliding Window Common Spatial Pattern for Enhancing Motor Imagery Classification in EEG-BCI. IEEE Trans. Instrum. Meas..

[9] Jin J., Miao Y., Daly I., Zuo C., Hu D., Cichocki A. (2019). Correlation-Based Channel Selection and Regularized Feature Optimization for MI-Based BCI. Neural Netw..

[10] Park Y., Chung W. (2020). Optimal Channel Selection Using Correlation Coefficient for CSP Based EEG Classification. IEEE Access.

[11] Chen J., Yi W., Wang D., Du J., Fu L., Li T. (2022). FB-CGANet: Filter Bank Channel Group Attention Network for Multi-Class Motor Imagery Classification. J. Neural Eng..

[12] Varone G., Boulila W., Driss M., Kumari S., Khan M.K., Gadekallu T.R., Hussain A. (2024). Finger Pinching and Imagination Classification: A Fusion of CNN Architectures for IoMT-Enabled BCI Applications. Inf. Fusion.

[13] Altaheri H., Muhammad G., Alsulaiman M., Amin S.U., Altuwaijri G.A., Abdul W., Bencherif M.A., Faisal M. (2021). Deep Learning Techniques for Classification of Electroencephalogram (EEG) Motor Imagery (MI) Signals: A Review.

[14] Schirrmeister R.T., Springenberg J.T., Fiederer L.D.J., Glasstetter M., Eggensperger K., Tangermann M., Hutter F., Burgard W., Ball T. (2017). Deep Learning with Convolutional Neural Networks for EEG Decoding and Visualization. Hum. Brain Mapp..

[15] Lawhern V.J., Solon A.J., Waytowich N.R., Gordon S.M., Hung C.P., Lance B.J. (2018). EEGNet: A Compact Convolutional Network for EEG-Based Brain-Computer Interfaces. J. Neural Eng..

[16] Hu J., Shen L., Sun G. Squeeze-and-Excitation Networks. Proceedings of the IEEE Conference on Computer Vision and Pattern Recognition (CVPR).

[17] Woo S., Park J., Lee J.-Y., Kweon I.S. CBAM: Convolutional Block Attention Module. Proceedings of the Proceedings of the European Conference on Computer Vision (ECCV).

[18] Hassanpour A., Moradikia M., Adeli H., Khayami S.R., Shamsinejadbabaki P. (2019). A Novel End-to-End Deep Learning Scheme for Classifying Multi-Class Motor Imagery Electroencephalography Signals. Expert. Syst..

[19] Tiwari A., Chaturvedi A. (2023). Automatic EEG Channel Selection for Multiclass Brain-Computer Interface Classification Using Multiobjective Improved Firefly Algorithm. Multimed. Tools Appl..

[20] Jindal K., Upadhyay R., Singh H.S. (2022). A novel EEG channel selection and classification methodology for multi-class motor imagery-based BCI system design. Int. J. Imaging Syst. Technol..

[21] Mahamune R., Laskar S.H. (2023). An Automatic Channel Selection Method Based on the Standard Deviation of Wavelet Coefficients for Motor Imagery Based Brain–Computer Interfacing. Int. J. Imaging Syst. Technol..

[22] Chen S., Sun Y., Wang H., Pang Z. (2020). Channel Selection Based Similarity Measurement for Motor Imagery Classification. Proceedings of the 2020 IEEE International Conference on Bioinformatics and Biomedicine (BIBM).

[23] Ingolfsson T.M., Hersche M., Wang X., Kobayashi N., Cavigelli L., Benini L. (2020). EEG-TCNet: An Accurate Temporal Convolutional Network for Embedded Motor-Imagery Brain–Machine Interfaces. Proceedings of the 2020 IEEE International Conference on Systems, Man, and Cybernetics (SMC).

[24] Wu H., Niu Y., Li F., Li Y., Fu B., Shi G., Dong M. (2019). A Parallel Multiscale Filter Bank Convolutional Neural Networks for Motor Imagery EEG Classification. Front. Neurosci..

[25] Musallam Y.K., AlFassam N.I., Muhammad G., Amin S.U., Alsulaiman M., Abdul W., Altaheri H., Bencherif M.A., Algabri M. (2021). Electroencephalography-Based Motor Imagery Classification Using Temporal Convolutional Network Fusion. Biomed. Signal Process. Control.

[26] Liu X., Xiong S., Wang X., Liang T., Wang H., Liu X. (2023). A Compact Multi-Branch 1D Convolutional Neural Network for EEG-Based Motor Imagery Classification. Biomed. Signal Process. Control.

[27] Salami A., Andreu-Perez J., Gillmeister H. (2022). EEG-ITNet: An Explainable Inception Temporal Convolutional Network for Motor Imagery Classification. IEEE Access.

[28] Jia H., Yu S., Yin S., Liu L., Yi C., Xue K., Li F., Yao D., Xu P., Zhang T. (2023). A Model Combining Multi Branch Spectral-Temporal CNN, Efficient Channel Attention, and LightGBM for MI-BCI Classification. IEEE Trans. Neural Syst. Rehabil. Eng..

[29] Zhang X., Miao Z., Menon C., Zheng Y., Zhao M., Ming D. (2023). Priming Cross-Session Motor Imagery Classification with a Universal Deep Domain Adaptation Framework. Neurocomputing.

[30] Kim D.-H., Shin D.-H., Kam T.-E. (2023). Bridging the BCI Illiteracy Gap: A Subject-to-Subject Semantic Style Transfer for EEG-Based Motor Imagery Classification. Front. Hum. Neurosci..

[31] Echtioui A., Zouch W., Ghorbel M., Mhiri C., Hamam H. (2023). Classification of BCI Multiclass Motor Imagery Task Based on Artificial Neural Network. Clin. EEG Neurosci..

[32] Abdullah, Faye I., Islam M.R. (2022). EEG Channel Selection Techniques in Motor Imagery Applications: A Review and New Perspectives. Bioengineering.

[33] Khan M.A., Lali M.I.U., Sharif M., Javed K., Aurangzeb K., Haider S.I., Altamrah A.S., Akram T. (2019). An Optimized Method for Segmentation and Classification of Apple Diseases Based on Strong Correlation and Genetic Algorithm Based Feature Selection. IEEE Access.

[34] Liu Z., Chang B., Cheng F. (2021). An Interactive Filter-Wrapper Multi-Objective Evolutionary Algorithm for Feature Selection. Swarm Evol. Comput..

[35] Maleki N., Zeinali Y., Niaki S.T.A. (2021). A K-NN Method for Lung Cancer Prognosis with the Use of a Genetic Algorithm for Feature Selection. Expert. Syst. Appl..

[36] Padfield N., Ren J., Murray P., Zhao H. (2021). Sparse Learning of Band Power Features with Genetic Channel Selection for Effective Classification of EEG Signals. Neurocomputing.

[37] Yang J., Singh H., Hines E.L., Schlaghecken F., Iliescu D.D., Leeson M.S., Stocks N.G. (2012). Channel Selection and Classification of Electroencephalogram Signals: An Artificial Neural Network and Genetic Algorithm-Based Approach. Artif. Intell. Med..

[38] Albasri A., Abdali-Mohammadi F., Fathi A. (2019). EEG Electrode Selection for Person Identification Thru a Genetic-Algorithm Method. J. Med. Syst..

[39] He L., Hu Y., Li Y., Li D. (2013). Channel Selection by Rayleigh Coefficient Maximization Based Genetic Algorithm for Classifying Single-Trial Motor Imagery EEG. Neurocomputing.

[40] Brunner C., Leeb R., Müller-Putz G.R., Schlögl A., Pfurtscheller G. (2008). BCI Competition 2008--Graz Data Set A. Inst. Knowl. Discov. (Lab. Brain-Comput. Interfaces) Graz Univ. Technol..

[41] Tragoudaras A., Antoniadis C., Massoud Y. (2023). Enhancing DNN Models for EEG/ECoG BCI With a Novel Data-Driven Offline Optimization Method. IEEE Access.

[42] Tragoudaras A., Fanaras K., Antoniadis C., Massoud Y. (2023). Data-Driven Offline Optimization of Deep CNN Models for EEG and ECoG Decoding. Proceedings of the 2023 IEEE International Symposium on Circuits and Systems (ISCAS).

[43] Li Y., Guo L., Liu Y., Liu J., Meng F. (2021). A Temporal-Spectral-Based Squeeze-and- Excitation Feature Fusion Network for Motor Imagery EEG Decoding. IEEE Trans. Neural Syst. Rehabil. Eng..

[44] Howard A.G., Zhu M., Chen B., Kalenichenko D., Wang W., Weyand T. (2017). Mobilenets: Efficient Convolutional Neural Networks for Mobile Vision Applications. arXiv.

[45] Ioffe S., Szegedy C. Batch Normalization: Accelerating Deep Network Training by Reducing Internal Covariate Shift. Proceedings of the 32nd International Conference on Machine Learning.

[46] Chaudhari S., Mithal V., Polatkan G., Ramanath R. (2021). An Attentive Survey of Attention Models. ACM Trans. Intell. Syst. Technol..

[47] Madhu G., Gajapaka S.M., Bharadwaj L. (2022). A Simple Attention Block Embedded in Standard CNN for Image Classification. Proceedings of the 2022 International Conference on Applied Artificial Intelligence and Computing (ICAAIC).

[48] Karmakar P., Teng S.W., Lu G. (2021). Thank You for Attention: A Survey on Attention-Based Artificial Neural Networks for Automatic Speech Recognition. arXiv.

[49] Lun X., Yu Z., Wang F., Chen T., Hou Y. (2021). A Novel Approach of CNN for Human Motor Imagery Recognition Using the Virtual Electrode Pairs. J. Intell. Fuzzy Syst..

[50] Roots K., Muhammad Y., Muhammad N. (2020). Fusion Convolutional Neural Network for Cross-Subject Eeg Motor Imagery Classification. Computers.

[51] Magboo V.P.C., Magboo M.S.A. (2021). Machine Learning Classifiers on Breast Cancer Recurrences. Procedia Comput. Sci..

[52] Long T., Wan M., Jian W., Dai H., Nie W., Xu J. (2023). Application of Multi-Task Transfer Learning: The Combination of EA and Optimized Subband Regularized CSP to Classification of 8-Channel EEG Signals with Small Dataset. Front. Hum. Neurosci..

[53] Jiang Q., Zhang Y., Zheng K. (2022). Motor Imagery Classification via Kernel-Based Domain Adaptation on an SPD Manifold. Brain Sci..

[54] Dai G., Zhou J., Huang J., Wang N. (2020). HS-CNN: A CNN with Hybrid Convolution Scale for EEG Motor Imagery Classification. J. Neural Eng..

[55] Saputra M.F., Setiawan N.A., Ardiyanto I. (2019). Deep Learning Methods for EEG Signals Classification of Motor Imagery in BCI. IJITEE (Int. J. Inf. Technol. Electr. Eng.).

[56] Santamaria-Vazquez E., Martinez-Cagigal V., Vaquerizo-Villar F., Hornero R. (2020). EEG-Inception: A Novel Deep Convolutional Neural Network for Assistive ERP-Based Brain-Computer Interfaces. IEEE Trans. Neural Syst. Rehabil. Eng..

